# Targeted depletion of *PIK3R2* induces regression of lung squamous cell carcinoma

**DOI:** 10.18632/oncotarget.13195

**Published:** 2016-11-08

**Authors:** Jesús Vallejo-Díaz, Manuel Olazabal-Morán, Ariel E. Cariaga-Martínez, María J. Pajares, Juana M. Flores, Ruben Pio, Luis M. Montuenga, Ana Clara Carrera

**Affiliations:** ^1^ Department of Immunology and Oncology, Centro Nacional de Biotecnología (CNB-CSIC), Universidad Autónoma de Madrid, Cantoblanco, Madrid; ^2^ Program in Solid Tumors and Biomarkers, Center for Applied Medical Research (CIMA), Pamplona, Spain; ^3^ Department of Animal Medicine and Surgery, School of Veterinary Medicine, Complutense University of Madrid, Spain; ^4^ Navarra Health Research Institute (IDISNA), University of Navarra, Pamplona, Spain; ^5^ Department of Histology and Pathology, School of Medicine, University of Navarra, Pamplona, Spain; ^6^ Department of Biochemistry and Genetics, School of Science, University of Navarra, Pamplona, Spain

**Keywords:** lung cancer, SQCC, PIK3R2, targeted therapy, PI3K pathway

## Abstract

Oncogenic mutations in the PI3K/AKT pathway are present in nearly half of human tumors. Nonetheless, inhibitory compounds of the pathway often induce pathway rebound and tumor resistance. We find that lung squamous cell carcinoma (SQCC), which accounts for ~20% of lung cancer, exhibits increased expression of the PI3K subunit *PIK3R2*, which is at low expression levels in normal tissues. We tested a new approach to interfere with PI3K/AKT pathway activation in lung SQCC. We generated tumor xenografts of SQCC cell lines and examined the consequences of targeting *PIK3R2* expression. In tumors with high *PIK3R2* expression, and independently of *PIK3CA, KRAS*, or *PTEN* mutations, *PIK3R2* depletion induced lung SQCC xenograft regression without triggering PI3K/AKT pathway rebound. These results validate the use *PIK3R2* interfering tools for the treatment of lung squamous cell carcinoma.

## INTRODUCTION

Lung cancer is one of the main causes of death worldwide [1–3]. There are two major lung cancer histological types: small cell lung cancer (SCLC) and non-small cell lung cancer (NSCLC); the latter comprising three subtypes: adenocarcinoma, squamous cell carcinoma (SQCC) and large cell lung carcinoma [4–6]. Approximately 20% of lung cancers are SQCC, and survival rates for lung SQCC remain low. Development of therapies for this tumor type is thus a major research objective [2].

In the last decade, the introduction of next-generation sequencing aided the detailed description of the lung cancer mutational profile. A variety of agents that target EGFR (epidermal growth factor receptor), ALK (anaplastic lymphoma kinase), or ROS1 (*c-ros* oncogene 1) have improved the outcome of lung cancer patients bearing specific driver mutations. These studies also provided a list of less frequent potential driver mutations, which could be relevant in a proportion of patients; a number of trials are currently testing new molecularly targeted compounds [2–9]. For NSCLC, however, effective targeted therapies have mainly improved treatment of adenocarcinoma [2]. The SQCC mutational profile is complex and distinct from other NSCLC. The most frequent alterations found are *TP53*, *NFE2L2, KEAP1*, several genes involved in squamous cell differentiation, *CDKN2A, RB1*, *FGFR1* amplifications, and other mutations at low frequencies; the class IA phosphoinositide 3-kinase (PI3K) is also deregulated in SQCC [4, 8, 10, 11].

PI3K are dimers formed by a catalytic subunit (p110α, p110β or the hematopoietic isoform p110δ, encoded by *PIK3CA*, *CB*, and *CD*) and a regulatory subunit (p85α, p85β, or the muscle-specific p55γ isoform, encoded by *PIK3R1*, *R2*, and *R3*, respectively) [12, 13]. PI3K catalyzes the formation of phosphatidylinositol (PI) 3,4,5-trisphosphate (PIP3), which activates a signaling cascade that involves protein kinase B (PKB o Akt), the target of rapamycin (TOR), etc; PIP3 levels are downregulated by the phosphatase PTEN. PI3K activation induces cell survival and contributes to promote cell cycle progression, cell migration and the cancer-associated metabolic switch [12, 13, 14]. Alterations in the PI3K pathway in SQCC include amplification of *PIK3CA* (3q.26.3), *PIK3CB* (3q.22.3) and *PIK3R2* (19q13.2-4) [10, 11, 14]. *PIK3CA* is altered in 15% of SQCC samples, and *PTEN* defects are found at similar frequency [8], making PI3K a promising candidate for targeted therapy.

Despite the immense effort to implement PI3K/Akt/mTOR inhibitors for clinical treatment of solid tumors, the results in some cases showed limited efficacy [15]. For NSCLC, the recently reported results for a clinical trial using the PI3K inhibitor buparlisib (a class I pan-PI3K inhibitor) showed that tumor responses were only found in 3% of SQCC and non-squamous NSCLC patients (selected to exhibit activation of PI3K pathway) [16, 17]. Treatment with these inhibitors often triggers ablation of inhibitory feedback pathways and activation of other receptors that cause pathway rebound and resistance [14, 15, 18–23].

In addition to *PTEN* mutation, or *PI3KCA/CB* amplification, PI3K activity is enhanced by mutations or deregulated expression of its regulatory subunits. We previously shown that p85β and p85α have non-redundant functions, a distinct subcellular localization, and a different pattern of expression in normal and transformed cells, p85α is more abundant in normal cells, whereas p85β levels is enhanced in melanoma, breast and colon cancer [24, 25]. p85β exhibits a higher affinity for the enzyme substrate (PI4,5P_2_); in addition, whereas p85α fully inhibits the activity of associated p110 and functions as a tumor suppressor, p85β/p110 show a residual activity in the absence of growth factors; in addition, p85β exhibits oncogenic activity [24, 26]. Although p85β overexpression accelerated tumor progression in the mouse [24], it was unknown whether depletion of p85β in an already developed tumor might induce tumor regression. Here we show that *PIK3R2* expression is increased in human lung SQCC, and its depletion induced SQCC tumor regression, supporting development of *PIK3R2* interfering tools as a therapy for lung SQCC.

## RESULTS

### Human lung squamous cell carcinoma cell lines express high *PIK3R2* levels

**Figure 1:**
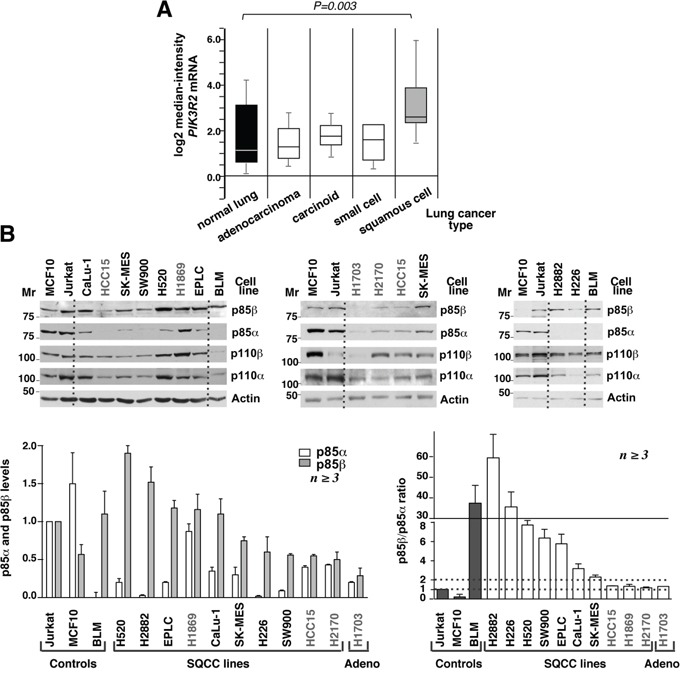
Human lung squamous cell carcinoma cell lines express high *PIK3R2* levels **A.** Comparison of *PIK3R2* mRNA expression in different lung cancer types (ref. 4, Bhattacharjee at al, 2001; www.oncomine.org). The study included 17 samples of normal lung, 132 adenocarcinomas, 20 lung carcinoids tumor, 6 small cell carcinomas and 21 lung SQCC. Data shown as mean ± SD, Student's *t* test. **B.** Cell lines were maintained in exponential growth, lysed, and lysates adjusted for equal protein loading. Extracts (50 μg) were resolved in SDS-PAGE and examined in western blot (WB) using the indicated antibodies. Mr, relative mobility. p85β signals in the different lanes were measured, normalized to the loading control and compared to the p85β signal in Jurkat cells considered 1; p85α signals was examined similarly and compared to the signal in Jurkat cells, also considered 1 as these cells contain comparable amounts of p85α and p85β. Graph (bottom left) shows the mean ± SEM of p85α and β signal normalized for that of Jurkat cells. To define the excess of p85β compared to p85α, we calculated the p85β/p85α signal intensity ratio (each compared to that of Jurkat cells). Graph at the bottom right shows the mean ± SEM of p85β/p85α ratios; SQCC lines with ratios <2 are in grey. Dashed lines indicate p85β/p85α ratios of 1 and 2.

The PI3K p85 regulatory subunit binds, stabilizes and induces activation of the p110 catalytic subunit [27]. Normal cells express higher levels of *PIK3R1* (which encodes p85α) than of *PIK3R2* (p85β) [24, 28, 29]; in contrast, metastatic melanoma, invasive breast cancer and advanced colon carcinoma show a marked increase in *PIK3R2* expression, which correlates with tumor grade [24, 25]. Analysis of gene expression data shows that *PIK3R2* expression is also frequently enhanced in lung SQCC (Figure 1A) [www.oncomine.org, Ref. 30][also Ref. 31–33].

We tested whether the increase in *PIK3R2* levels was also observed in lung SQCC cell lines and translates into enhanced p85β protein expression. We determined p85α and p85β levels as well as those of p110α and p110β catalytic subunits in ten lung SQCC cell lines (described in Supplementary Table S1). As controls, we used an adenocarcinoma line (H1703), a normal epithelial cell line (MCF10), and an advanced melanoma cell line (BLM) with increased p85β expression [25]. In addition, we included the human T cell lymphoblastoid Jurkat cell line (JK), which contains similar levels of p85α and p85β [29]. We gave a value of 1 to the signal intensity of p85α and of p85β in JK cell lanes, and refer the signal intensity of the different lines to that of JK cells (considered 1). In addition, we determined the relative p85β/α content by calculating their ratio in each cell line compared to that of JK cells (which has a ratio of 1).

SQCC lines expressed p110α and p110β isoforms; since p85 protects p110 from degradation [27], cells with higher p85 levels also had increased p110 levels (Figure 1A). In the case of regulatory subunits, MCF10 epithelial cells had higher p85α than p85β levels; the H1703 adenocarcinoma, and three of the ten SQCC lines (HCC15, H1869, H2170) had similar p85α and p85β levels; the remaining seven SQCC lineshad higher levels of p85β, with p85β/p85α ratios varying from 2 to >10 (Figure 1B). Thus, both lung SQCC tumors and more than 50% of the SQCC cell lines preferentially expressed *PIK3R2*.

### *PIK3R2* depletion reduces PI3K pathway activation in lung SQCC cells

**Figure 2:**
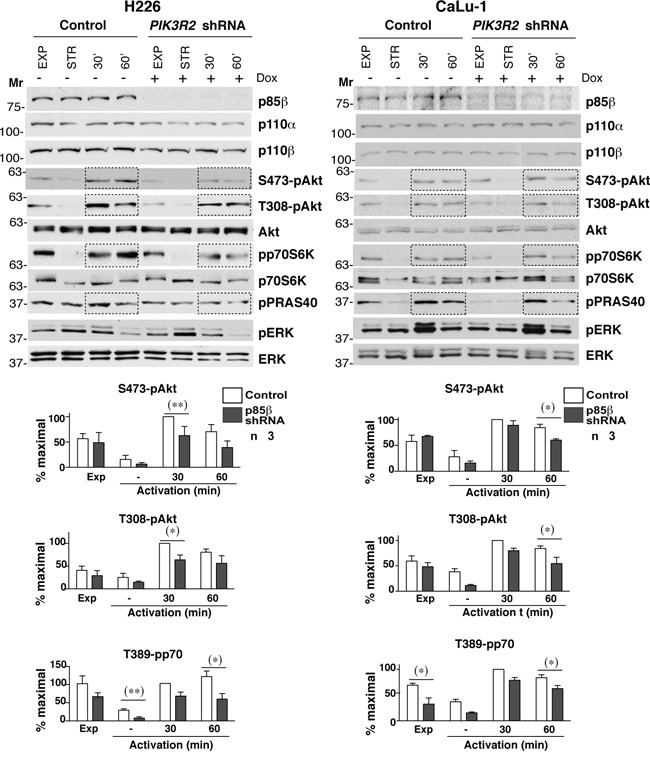
*PIK3R2* depletion reduces PI3K pathway activity in CaLu-1 and H226 lung SQCC cell lines CaLu-1 or H226 cells expressing inducible *PIK3R*2 shRNA were cultured alone or with doxycycline (2 μg/ml, 72 h); extracts were examined in WB. Mr indicates relative mobility. Graphs show the percent of maximal S472-pAkt, T308-pAkt and T389-pp70S6K signal, normalized to Akt or p70S6K and compared to the control cell signal at 30 min post-stimulation, considered 100%. * P<0.05; ** P<0.01; Student's *t* test.

The expression of p85β/p110α complexes in NIH3T3 cells enhances basal PI3K pathway activation (in the absence of growth factors) [24]. Considering that SQCC shows greater p85β levels compared to p85α, we tested the result of reducing *PIK3R2* expression on PI3K pathway activityin two representative SQCC cell lines, H226 (p85β/p85α ratio >10) and CaLu-1 (p85β/p85α ratio ~3). We cloned a short hairpin *PIK3R2*-specific sequence in an inducible lentiviral vector (pLKO-TetON) and obtained stable clones that expressed *PIK3R2* shRNA after doxycycline induction (72 h) (Figure 2). Cells were examined in exponential growth, after serum starvation (2 h), and after 30 or 60 min stimulation with serum. The majority of the regulatory form in H226 cells is p85β, and its depletion moderately reduced p110β and p110α levels; this effect was not as evident in CaLu-1 cells, which suggests that the remaining p85α in these cells is sufficient to stabilize p110 (Figure 2).

To determine the activation state of PI3K pathway, we examined phospho-Akt (pS473-and pT308-Akt) levels, the Akt substrate PRAS40 (pT246-PRAS40) and the mTOR substrate p70S6K (pT389-p70S6K); we also examined the MAPK pathway status (T202/T204-pErk). In H226 cells, *PIK3R2* depletion reduced pAkt, pPRAS40 and p70S6K, with no notable effect on pErk (Figure 2). In CaLu-1 cells, pAkt levels were lower than in H226 cells; nonetheless, *PIK3R2* knockdown also reduced pS473-Akt, pT308-Akt, pPRAS40 and pT389-p70S6K levels, mainly at 1 h post-activation (Figure 2).

In a similar assay, *PIK3R1* silencing did not markedly affect pErk levels (a moderate reduction was seen in H226). In addition, p85α depletion reduced p110β and p110α levels in both cell lines (more clearly in CaLu-1), which suggests that p85α more effectively protects p110 from degradation than p85β; activation of most PI3K effectors were either unaffected (pPRAS40) or increased (T308-pAkt, T389-pp70S6K) after p85α depletion (Supplementary Figure S1), with the exception of S473-pAkt in CaLu-1 cells, which appeared to require both p85β and p85α expression for optimal S473-pAkt phosphorylation (Figure 2). This finding might be explained by the p85α contribution to p110 stability. The increased activation of the remaining PI3K effectors after *PIK3R1* silencing, despite reduction of p110 levels, suggests that p85α depletion causes enrichment in p85β/p110 complexes (Figure 2), which enhance PI3K activation [24]. These observations show that p85β is the main regulatory isoform that mediates PI3K pathway activation in H226 and CaLu-1 lung SQCC cells.

### *PIK3R2* depletion triggers SQCC apoptosis and xenograft regression

In breast and colon cancers *PIK3R2* overexpression correlates with tumor grade [24]. As *PIK3R2* expression is increased in SQCC tumors, we postulated that interference with *PIK3R2* in an established SQCC tumor would halt tumor progression. To examine the effect of reduced *PIK3R1* or *R2* expression in lung SQCC tumor xenografts, we infected different cell lines with an inducible lentiviral vector that encodes a short hairpin specific for *PIK3R1* or *R2*; stable clones expressed these shRNA after doxycycline induction. We optimized protocols for xenografts of the different cell lines in immunodeficient mice (see Methods); as controls we used SQCC cell lines expressing the pLKO empty vector (doxycycline-treated) or cell lines expressing *PIK3R1/2* shRNA (without doxycycline).

**Figure 3:**
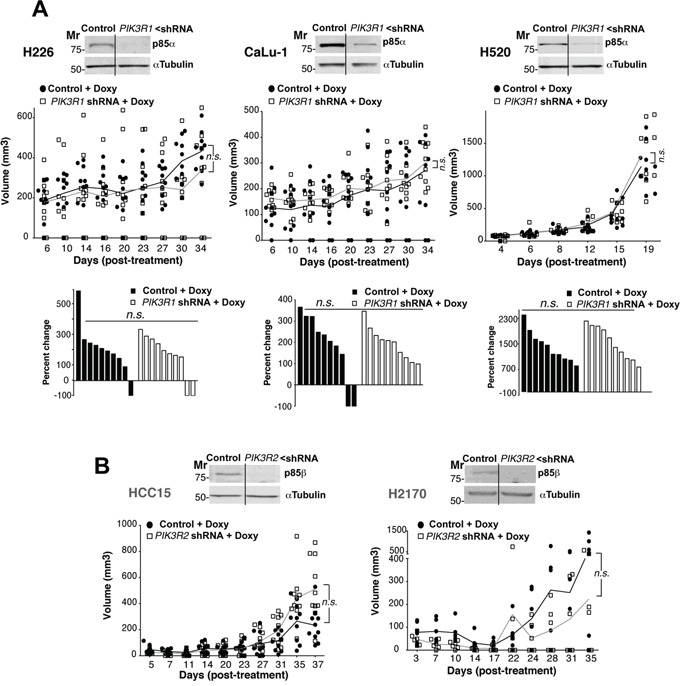
*PIK3R1* depletion does not trigger lung SQCC xenograft regression **A.** H226, CaLu-1 and H520 lung SQCC cell lines expressing inducible *PIK3R1* shRNA were cultured (72 h) and extracts examined in WB to confirm *PIK3R1* shRNA efficiency. Cell lines were expanded in culture and injected subcutaneously into *scid/scid* mice (~10^7^ cells in 100 μl PBS plus 100 μl matrigel). Graph (top) illustrates the size of each tumor at different times after initiation of treatment; n.s., not significant, two-way ANOVA test. Graph (bottom) shows percent change in size from beginning to end of treatment; n.s., not significant, Chi square test. Mr, relative mobility. **B.** Lung SQCC cell lines SK-MES and EPLC, with similar p85β and p85α levels, were infected with viruses encoding inducible control or *PIK3R2* shRNA; clones were selected and xenografts established in *scid/beige* as above. We compared tumor growth after doxycycline addition to drinking water. In WB, we tested *PIK3R2* shRNA efficiency in reducing p85β levels. The graph indicates the size of each tumor at different times after initiation of treatment. Differences between control and treated tumors was analyzed using a 2-way ANOVA test; n.s., not significant.

We first tested the effect of *PIK3R1* silencing on the growth of tumor xenografts derived from H226, H520, and CaLu-1 cells with p85β/p85α ratios >2. In response to doxycycline, the cells showed reduced *PIK3R1* levels, although this treatment did not significantly reduce tumor size (Figure 3A; Supplementary Figure S2A). We also analyzed the consequences of *PIK3R2* depletion in tumors of cells with a p85β/p85α ratio of ~1. In HCC15, H2170, *PIK3R2* depletion did not affect tumor size (Figure 3B; Supplementary Figure S2B). H1869 cells were resistant to lentiviral infection and could not be examined.

**Figure 4:**
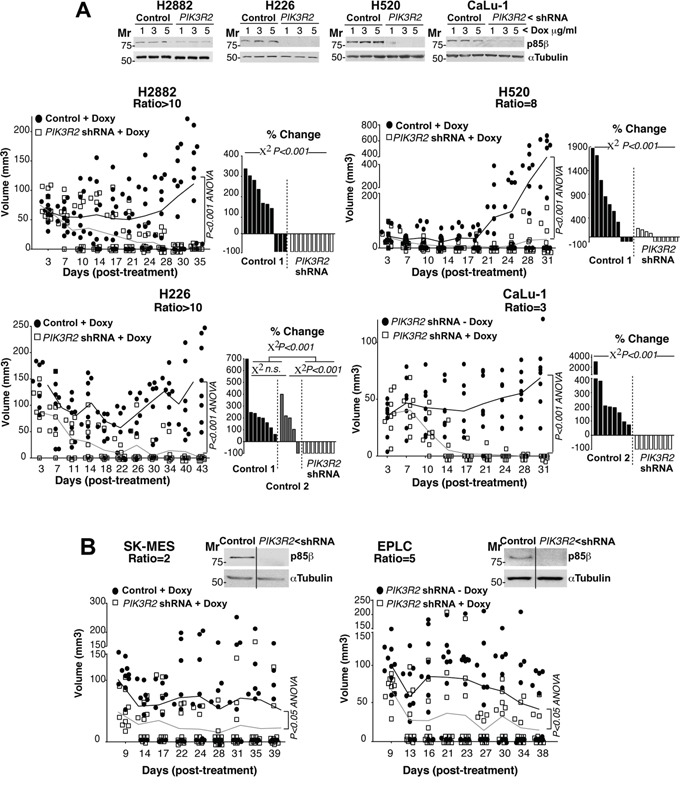
*PIK3R2* depletion induces SQCC xenograft regression **A, B.** Lung SQCC cell lines expressing inducible *PIK3R2* shRNA were cultured (72 h) at indicated doxycycline concentrations. Sample extracts were examined in WB to confirm *PIK3R2* shRNA efficiency in reducing p85β levels (top). Cell lines were expanded in culture and injected subcutaneously into *scid/beige* mice (~10^7^ cells in 100 μl PBS plus 100 μl matrigel). Tumors developed for several days until they reached ~50 mm^3^; we then included doxycycline (2 mg/ml) in drinking water and measured tumor size three times/week. We compared tumor growth after doxycycline addition to drinking water. As a control, we used tumors generated with cell lines expressing empty vector and treated with doxycycline (control 1) or generated with cell lines expressing inducible *PIK3R2* shRNA with no doxycycline (control 2); both controls gave comparable results. Graphs in A, (left) and B) indicate the size of each tumor at different times after treatment. Differences between control and treated mouse tumors were analyzed with a 2-way ANOVA test. Graphs in A, (right) show percent change in tumor size, comparing the final with initial tumor size (pre-treatment). We compared groups using the Chi square test.

We next analyzed the effect of depleting *PIK3R2* in tumor xenografts from lung SQCC cells with preferential *PIK3R2* expression. Seven of the ten SQCC cell lines exhibited increased expression of *PIK3R2*. All of them were analyzed, except for SW900 cells, which showed extremely low division rates that made it difficult to select clones expressing *PIK3R2* shRNA. We detected two types of response after *PIK3R2* depletion, tumors derived from four lines were almost completely eliminated (H2882, H520, H226, CaLu-1; Figure 4A, Supplementary Figure S3A), whereas tumor from the other two lines (SK-MES-1, EPLC-272H) showed reduced growth, although the response was not as marked (Figure 4B, Supplementary Figure S3B). The response to *PIK3R2* depletion was detected in SQCC cell lines with predominant *PIK3R2* expression, but it was not proportional to the p85β/p85α ratio, and did not correlate with *PTEN, KRAS* or *PIK3CA* mutation or with higher *PIK3CA* or *PIK3CB* expression (Figure 4, Supplementary Table S2). For SK-MES-1 and EPLC, results were similar in *scid/scid* mice and *scid/beige* mice, which lack NK cells [34] (Figure 4), excluding an effect of NK cells in the responses.

**Figure 5:**
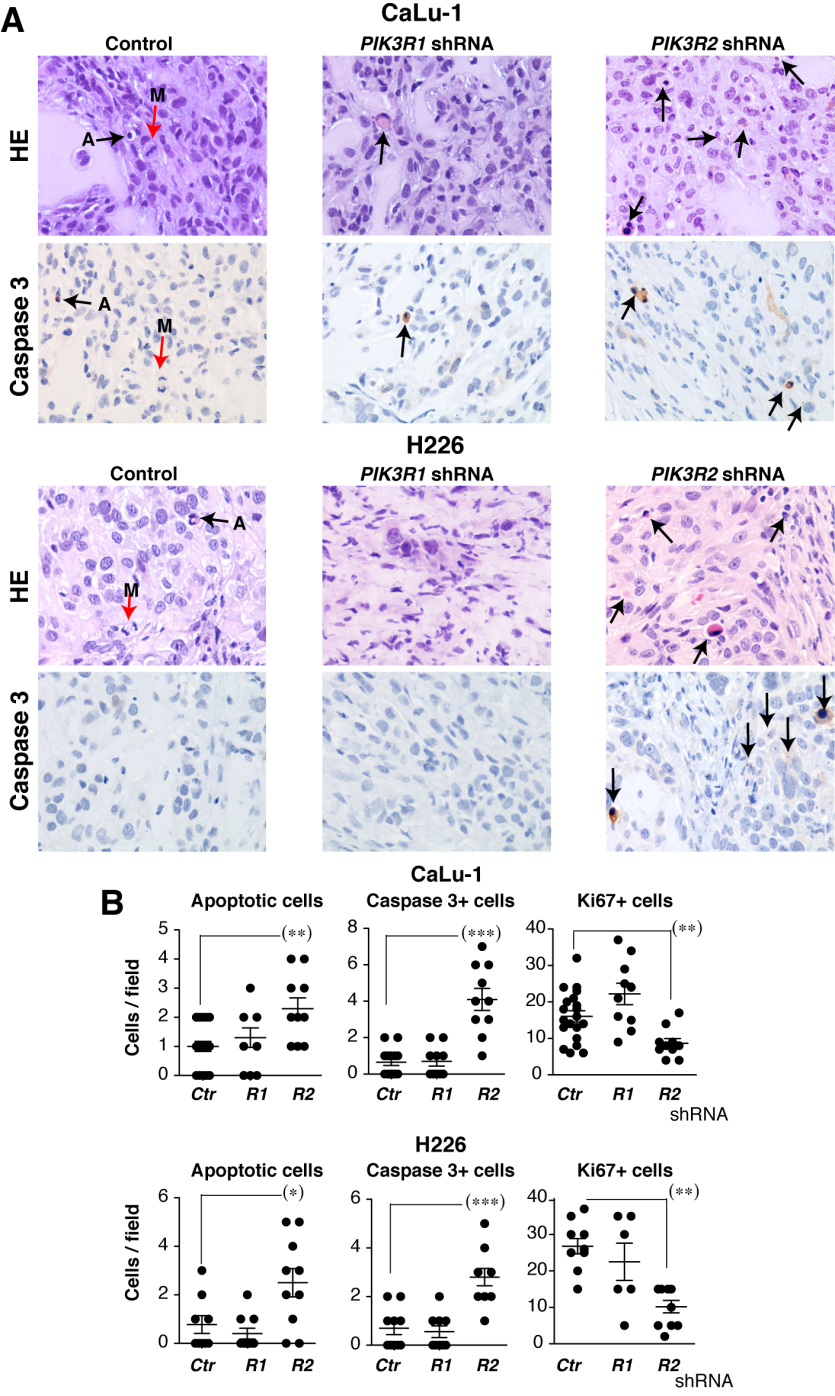
*PIK3R2* depletion induces cell death in SQCC xenografts Tumor xenografts were obtained using H226 and CaLu-1 cells, expressing inducible *PIK3R1* or *PIK3R*2 shRNA. When tumors reached ~50 mm^3^, mice were treated with doxycycline in drinking water (2 mg/ml). Mice were sacrificed when tumors size began to diminish. **A.** Histological examination of representative tumors and staining of active caspase 3 in control, p85α and p85β shRNA-treated samples (40x). Arrows show mitotic (M) and apoptotic cells. **B.** Graphs show the number of apoptotic, caspase 3+ or Ki-67+ cells per field (40x) in tumors treated with indicated shRNA. *** P <0.001; ** P<0.01; * P <0.05; Student's *t* test.

To examine the effect of reducing *PIK3R2* levels at a cellular level, we prepared a set of xenografts from H226 and CaLu-1 cells and mice were sacrificed at experiment midterm, when the tumor size began to diminish. Histological examination of these tumor samples showed a low mitotic index (Figure 5, red arrows), which was even lower after *PIK3R2* depletion. Few apoptotic figures were observed in control or *PIK3R1* shRNA-treated tumors, and were numerous in *PIK3R2*-depleted tumors (Figure 5). Immunostaining of Ki-67+ and active caspase 3 in control, *PIK3R1* and *PIK3R2* shRNA-treated samples showed a lower number of dividing cells and a larger number of apoptotic cells after *PIK3R2* shRNA treatment (Figure 5). These results indicate that *PIK3R2* depletion in SQCC lines decelerates cell growth and induces cell death.

**Figure 6:**
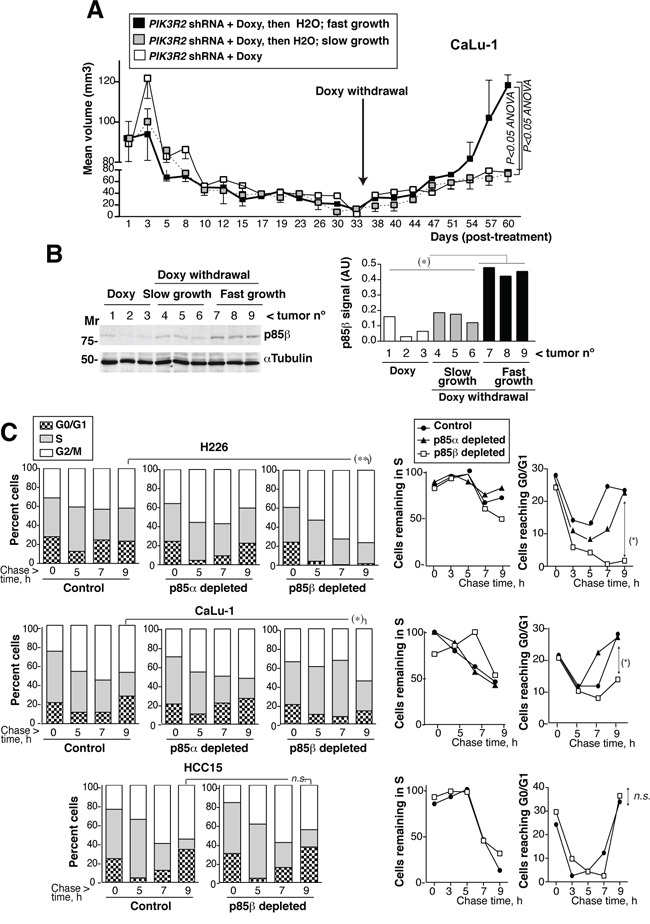
*PIK3R2* reconstitution restores SQCC xenograft growth; *PIK3R2* depletion decelerates cell cycle progression **A.** Xenografts were established using CaLu-1 cells expressing inducible-*PIK3R2* shRNA. When tumors reached a volume ~100 mm^3^, mice were treated with doxycycline for ~one month, when we randomly divided the animals in two groups. Doxycycline was withdrawn in one of the groups and maintained in the other. Graph shows the Mean ± SEM size of tumors kept in doxycycline (white square) as well as those deprived of the treatment, which were divided in slow- (grey) or fast-growing tumors (black). Differences between groups were analyzed using a 2-way ANOVA test. **B.** WB confirmed p85β re-expression in fast growing tumors and p85β low levels in the slow-growth tumors. Graph shows p85β signal intensity (corrected for the loading control) in arbitrary units (A.U.). Mr. indicates relative mobility. C. CaLu-1 or H226 cells expressing control, *PIK3R*1 or *PIK3R*2 shRNA were cultured alone or with doxycycline (5 μg/ml, 72 h). A non-responsive cell line (HCC15, control) expressing *PIK3R*2 shRNA was treated similarly. Cells were labeled with BrdU (20 μM, 1h; 90 min in the case of H226) then deprived of BrdU and chased at different times. We examined the percentage of BrdU+ cells that progressed from 2N (G0/G1) to 2-to-4N (S) and to 4N DNA content (G2/M phases) by flow cytometry (Mean percentage, *n* = 3)(left panels). In the medium panel, we represent the proportion of cells remaining in S phase at different times compared to maximal, considered 100%. Right panels show the percentage of cells in G0/G1 at different times. *P<0.05, differences between cell cycle distribution were examined using Chi square test; percent of S or G0/G1 cells was compared using Student's *t* test.

In one of the cell lines, we tested whether rescue of p85β expression restores tumor growth; we also tested the efficacy of a prolonged treatment. We used CaLu-1 cells expressing doxycycline-inducible *PIK3R2* shRNA. In this assay, we allow the tumors to grow until ~100mm^3^, when we then began the treatment with doxycycline in the drinking water (2 mg/ml). The tumor size reduction was slower than when treating smaller tumors (Figure 6A ~100mm^3^ *versus* Figure 4A ~50 mm^3^ tumors), which suggests that accessibility to doxycycline in big tumors might be suboptimal and the treatment reduced tumor size more gradually. At ~1 month of treatment, most of the tumors were very small; animals were randomly divided in two groups, one was maintained and the other deprived of doxycycline. All the tumors maintained with doxycycline remained small for another month, although they show a slow but continuous moderate increase in size (Figure 6A). Among tumors deprived of doxycycline we detected two clearly distinct behaviors, one set remained small and behaved similarly than tumors maintained in doxycycline, whereas the other set grew at significantly higher rates and sizes (Figure 6A). The tumors with a marked regrowth index corresponded to those that restored p85β expression, while the set of tumors with moderate growth expressed very low p85β levels (Figure 6B). These results suggest that tumors remain responsive after a two-month treatment, although p85β depletion is not sufficient for a complete and stable tumor disappearance. In addition, restoration of p85β expression, results in marked tumor regrowth (Figure 6A,B).

In conclusion, *PIK3R2* depletion reduced growth of all SQCC tumors with enhanced p85β levels and was sufficient to trigger tumor regression in the six SQCC lines with this phenotype (Figure 4). Shrinkage of SQCC-derived tumors supports development of an approach aimed to decrease *PIK3R2* action for the treatment of SQCC.

### *PIK3R2* depletion decelerates cell cycle progression

We also examined the effect on cell cycle progression of depleting *PIK3R2* in responsive (H226 and CaLu-1) and non-responsive (HCC15) cells. We BrdU-labeled newly synthesized DNA in exponentially growing cells, remove BrdU and collected the cells at various time points after BrdU deprivation (chase), to follow progression of BrdU+ S phase cells through cell cycle. This method permits examination of cell cycle in asynchronous cultures since tumor cells are difficult to synchronize in G0/G1 phases. The proportion of H226 cells labeled with BrdU during the 90 min pulse period was similar in control and *PIK3R1-*depleted cells but was approximately 30% lower in *PIK3R2-*depleted cells, supporting a slower G0/G1 transition to S phase. In addition, whereas the majority of control H226 cells made the S> G2/M transition similarly than *PIK3R1-*depleted cells and exited from G2/M to G0/G1 similarly (moderately faster in the case of control), *PIK3R2-*depleted cells entered G2/M and were accumulated in this phase without showing progression to G0/G1 phase at ~ 9h (Figure 6C). H226 cells have a p85β/p85α ratio >10; this behavior might reflect the need of a p85 molecule to complete cytokinesis [35]. In CaLu-1 cells (with a p85β/p85α ratio of ~3), cells were not sequestered in G2/M phase, possibly due to the presence of p85α; nonetheless, *PIK3R1* depletion moderately accelerated and *PIK3R2* depletion significantly decelerated cell cycle progression, as estimated by the rate of S phase exit and return to G0/G1 phases (Figure 6C). *PIK3R2* depletion did not affect S to G2/M transition, or exit from G2/M to G0/G1 in HCC15 cells (Figure 6C). This shows that p85β depletion selectively decelerates cell cycle progression in *PIK3R2* shRNA sensitive cells.

### *PIK3R2* depletion does not induce PI3K pathway rebound

**Figure 7:**
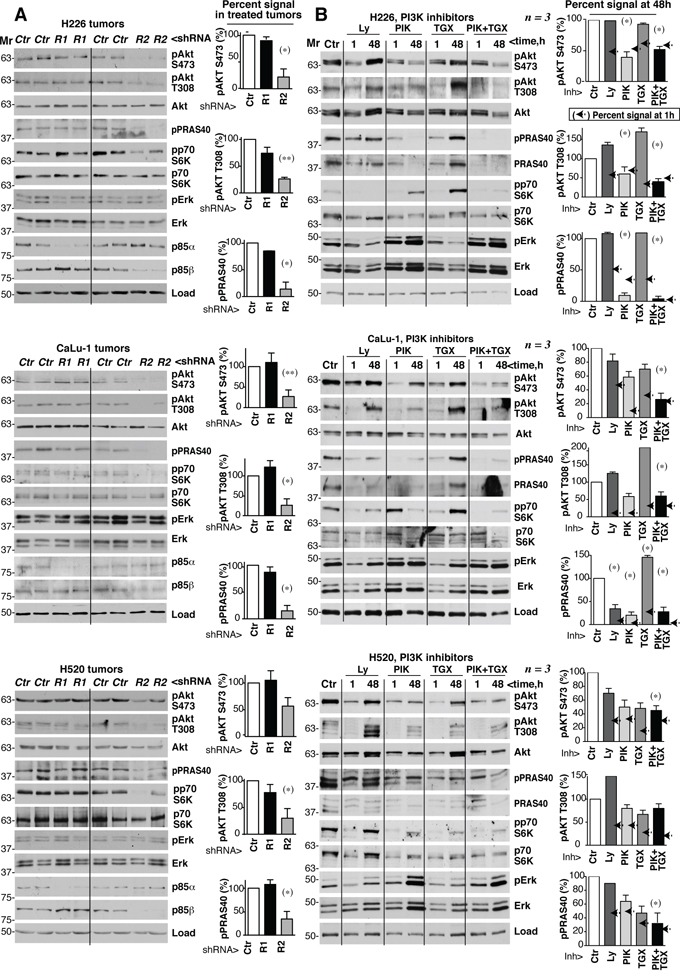
*PIK3R2* depletion, but not PI3K inhibitors, induces stable PI3K pathway inhibition **A.** Tumor xenografts were obtained using H226, CaLu-1 and H520 lung SQCC cells expressing inducible *PIK3R1* or *PIK3R*2 shRNA. When tumors reached ~50 mm^3^, mice were treated with doxycycline in drinking water (2 mg/ml); they were sacrificed when tumors began to diminish. Normalized tumor extracts were examined by WB with indicated antibodies. **B.** H226, CaLu-1 and H520 cells were cultured in exponential growth were incubated with: vehicle (DMSO, 1:10^3^ V:V), Ly294002 (5 μM), PIK75 (200 nM), TGX221 (30 μM), or PIK75 (200 nM) plus TGX221 (30 μM) for the last 1 h of culture or during 48 h; extracts were tested in WB. Mr indicates relative mobility. In both A and B, the pAkt or pPRAS40 signal was measured and normalized to the loading control (Akt or tubulin, respectively), and compared to that in controls (DMSO, considered 100%). Graphs show percentage of signal compared to maximal as mean ± SD at 48h of treatment. Arrows on the right side of bars show the percentage of signal compared to maximal detected after 1h of treatment.* *P* <0.05; **P <0.01; unpaired Student's *t* test with Welch correction.

To analyze the status of PI3K pathway in shRNA-treated tumors, we tested extracts from control, *PIK3R1* and *PIK3R2* shRNA-treated-H226, -CaLu-1 and -H520 tumor samples. Depletion of *PIK3R2*, but not of *PIK3R1*, reduced pT308-, pS473-Akt, pPRAS40 and pp70S6K levels without notably affecting pErk/Erk levels (Figure 7A). Physiological PI3K activation is followed by induction of negative feedback mechanisms that maintain the transient nature of PI3K activation [12–22]. A major problem of PI3K inhibitor-based therapies is that they also abrogate these feedback inhibitory mechanisms. The combined effect of reducing enzyme as well as feedback pathways activities often results in net pathway reactivation after a few hours [12–22]. In some cases, this effect is observed with p110α inhibitors alone and is corrected by the combined addition of p110α plus p110β inhibitors; in other models, even pan-PI3K inhibitors induce pathway reactivation after long incubation periods [36–38]. Prolonged *PIK3R2* shRNA treatment induced stable PI3K pathway inhibition (Figure 7A), which might be the result of a net reduction in PI3K molecules levels that are stabilized by p85, or might indicate that negative feedback pathways do not act in lung SQCC lines. To determine whether *PIK3R2* deletion is advantageous compared to PI3K inhibition, we tested whether the inhibitors induce PI3K pathway reactivation after extended treatment. We optimized the dose of these compounds used for short-term inhibition of PI3K effectors; we tested rapamycin, a pan-PI3K inhibitor (Ly294002, Ly), a p110β-specific inhibitor (TGX221, TGX) and a p110α inhibitor (PIK75, PIK) [39]. Rapamycin only inhibited the mTOR substrate pS6K, while PIK and TGX reduced pAkt and pp70S6K levels in a dose-dependent manner (Supplementary Figure S4).

We tested whether prolonged treatment with Ly, TGX, PIK or a TGX/PIK combination could reactivate PI3K effectors. In H226 cells, treatment with optimal Ly or TGX doses for 48 h increased pAkt and ppPRAS40 levels compared to the 1 h treatment; although PIK or TGX/PIK maintained low pAkt and ppPRAS40 levels after 48 h, they increased total Erk and pErk levels (Figure 7B). In H520 and CaLu-1 cells, all inhibitors failed to restrain the pathway after incubation for 48 h, as estimated by measuring levels of PI3K effectors phosphorylation, which were higher at 48 h compared to 1 h treatment (Figure 7B). In H520 and CaLu-1 cells, we also detected a compensatory increase in pErk and total Erk levels after incubation with PIK and TGX/PIK (Figure 7B). Not all effectors recovered equally in the different treatments and cell lines. For instance, the TGX/PIK combination was more efficient that either inhibitor alone in maintaining low pathway activity, although reactivation was detected at 48 h compared to 1 h in H520 and CaLu-1 cells. Decreased pPRAS40 levels were paralleled by a reduction in PRAS40 cell levels and PIK treatment blocked pS6K phosphorylation less effectively in CaLu-1 than in the other cell lines, but showed longer duration (Figure 7B). Together, in most PI3K effectors examined, we detected an increase in phosphorylated forms after 48 h of incubation compared to 1 h (Figure 7B). In contrast to *PIK3R2* depletion, which stably inhibited the PI3K pathway, sustained treatment with PI3K inhibitors resulted in PI3K pathway reactivation.

## DISCUSSION

Targeted therapies are improving clinical management of lung adenocarcinoma, however, they have been less efficient for lung squamous cell carcinomas, which make up one-fifth of lung cancers. Increased expression of *PIK3R2*, which encodes the regulatory subunit p85β, enhances basal PI3K pathway activation and parallels tumor progression in melanoma, colon cancer and breast carcinoma [24, 25]. In the present study, we show that SQCC tumors also exhibit an increased *PIK3R2* expression. Moreover, we demonstrate that the interference with *PIK3R2* expression in established SQCC xenografts reduces tumor survival. In particular, interference with *PIK3R2* expression triggered tumor shrinkage in all the cell lines with predominant p85β expression. *PIK3R2* deletion induced cell death and stably inhibited the PI3K pathway, without inducing pathway rebound. Greater sensitivity of some tumors to *PIK3R2* depletion was independent of *PIK3CA* or *CB* expression levels and of *KRAS, PTEN* and *PIK3CA* mutation. These results support the development of therapies oriented to reduce the action of *PIK3R2* for lung SQCC.

Progress in whole-genome characterization has allowed generation of comprehensive profiles of genetic alterations in SQCC and identification of new therapeutic targets [2–11]. In addition to targets suggested by specific SQCC somatic DNA modifications, altered expression of WT proteins is also considered for clinical management. This is the case of MET tyrosine kinase receptor overexpression, which was observed in 29% of lung SQCC patients [2, 40]. Immunotherapy is another promising lung cancer treatment [41]. Nonetheless, the poor prognosis of lung cancer, and of SQCC in particular, encourages development of alternative strategies.

One of the most promising targets in oncology is PI3K. *PIK3CA* copy number gains are present are higher frequencies in SQCC than in lung adenocarcinoma [8]. Expectations are high for PI3K inhibitors in cancer. Nonetheless, although results are encouraging for PI3Kδ inhibitors in some hematopoietic tumors [ 14], the results from a recent clinical trial using buparlisib (a pan-PI3K inhibitor) for NSCLC patients (preselected to exhibit PI3K pathway activation) showed that only 3% (SQCC and non-squamous) responded by reducing tumor size [16, 17]. This is not exceptional, and except for some combined therapies that include PI3K, Akt, mTOR inhibitors, e.g., for breast and prostate cancer subtypes [36, 37, 42], treatment of solid tumors with these inhibitors has been often ineffective [12, 16, 17, 42, 43]. Better patient selection criteria [16] and identification of the pathway rebound and resistance mechanisms [22] have increased interest in combined therapy.

As commented above, deregulated WT gene expression (as is the case of MET receptor) has also been targeted successfully for therapy [2]. Considering that SQCC presents amplification of the chromosome region including *PIK3R2*, which encodes p85β [10, 11] and the observation that *PIK3R2* expression is often increased in clinical SQCC (Figure 1), we proposed that *PIK3R2* could be a promising target for lung SQCC. We based this proposal on the previous observation that p85β and p85α exhibit a distinct subcellular localization and a different pattern of expression in normal and transformed cells (p85α is the more abundant isoform in normal cells, whereas p85β expression is enhanced some tumors) [24, 25]. At the molecular level, p85β exhibits higher affinity for the enzyme substrate (PI4,5P_2_) than p85α, and a lower capacity to restrain p110 activity in the absence of growth factors[24]. In agreement with these features, p85β overexpression but not that of p85α induced cell transformation [24, 44]. Finally, although it was unknown whether depletion of p85β in an already developed tumor might induce tumor regression, *pik3r2*-deficient mice exhibit reduced colon cancer formation whereas p85β overexpression accelerated tumor progression in the mouse [24]. Here we show that *PIK3R2* depletion, but not that of *PIK3R1*, induced regression of established SQCC tumors exhibiting high *PIK3R2* levels (Figure 3 and 4). Several new avenues could be pursued in order to further dissect the differential function of p85α or p85β in cancer. First, the analysis of their differential regulation of the expression, examination of p85α or p85β associated proteins pre- and on-treatment and finally to ascertain the differential protein binding to the N-terminal region (the less conserved region) of each of the two proteins.

*PIK3R2* depletion in SQCC tumors with high *PIK3R2* expression caused sustained PI3K inhibition without inducing pathway reactivation (Figure 4 and 7), which would reduce the probability of resistance, an important drawback of enzyme inhibitors [13–16, 19–23, 35, 38]. The absence of pathway reactivation might be explained by the elimination after *PIK3R2* depletion of one of the targets of the feedback inhibitory pathways, the p85 molecule [38]. In a clinical setting, p85β expression level could potentially be examined in circulating cancer cells. Although tumor response to *PIK3R2* depletion required enhanced *PIK3R2* expression, the magnitude of the *PIK3R2* silencing effect in different tumor cells was not strictly proportional to the p85β expression levels, suggesting that other tumor features modulate this response. Nonetheless, tumor response to *PIK3R2* depletion did not require *PIK3CA, PTEN* or *KRAS* mutation, and caused shrinkage of all tumors examined with enhanced p85β expression. We show that tumors remain responsive after a two-month treatment, although p85β depletion was not sufficient for a complete and stable tumor disappearance (Figure 6). Rescue of p85β expression, resulted in tumor regrowth.

We have tested the consequences of depleting *PIK3R2* only in lung SQCC lines; it is possible that other tumor types showing enhanced *PIK3R2* expression at advances phases, such as breast and colon carcinoma or melanoma [24, 25], might also benefit from a therapeutic strategy based on *PIK3R2* depletion. One of the alterations found in SQCC is *PIK3CA* mutation or amplification [8]. Although any of the tested cell lines had *PIK3CA* mutations (Supplementary Table S2), CaLu-1 cells exhibit a K-Ras mutation that enhances PI3Kα activity [45] and responded to *PIK3R2* deletion. Future studies will definitively conclude on whether or not *PIK3CA* alteration affects p85β-dependence in lung SQCC.

The strength of antisense or interfering RNA gene expression regulators has been demonstrated extensively [46, 47], although their use as therapeutic tools in patients has been hampered by difficulty in delivering the RNA to the tumor; this might be facilitated in tumors accessible by aerosol therapy such as lung SQCC [46, 47]. The use of small molecules that impair p85β/p110 complex formation might also be an alternative; we have developed a FRET-based screening assay to search for such compounds (not shown). Finally, the CRISP-Cas approach, which has been successful in mice [48], might also be useful for *PIK3R2* deletion in lung SQCC with enhanced *PIK3R2* expression.

p85α:p85β ratios could be examined in the clinic by quantitative PCR since *PIK3R2* mRNA levels are proportional to p85 protein content [ref 29]; also, current techniques for gene copy number variation analysis could be applied to identify the SQCC cases with increased *PIK3R2* containing region. Additionally, p85α:p85β expression ratio might be examined in patient biopsy samples either by simultaneous analysis of the expression of both proteins by highly specific antibodies to the human proteins or by reverse phase protein analysis (RPPAs) applied to formalin-fixed and paraffin embedded (FFPE) tissue.

Although further technical improvements are needed, several siRNA targets and antisense oligonucleotides are currently under study and hold promise for the incorporation of these therapies for medical applications [49–52]. Their development for *PIK3R2* inhibition is justified by the clear potential of *PIK3R2* depletion for treatment of lung SQCC tumors as it induced tumor regression without triggering PI3K pathway reactivation.

## MATERIALS AND METHODS

### Cell culture

We used ten lung squamous cancer cell lines. Seven were purchased from the American Type Culture Collection (CaLu-1 (HTB-54), SK-MES-1 (HTB-58), H1869 (CRL-5900), H520 (HTB182), H2170 (CRL-5928), H226 (CRL-5826), SW900 (ATCC HTB-59) and two from the DSMZ collection (HCC-15 (ACC-496), EPLC-272H (ACC-383)). We also used the NSCLC line NCI-H2882 [53]. As controls, we used a human leukemic T cell lymphoblastoid line (Jurkat, TIB152), a non-tumorigenic breast epithelial cell line (MCF10, CRL10781) and a lung adenocarcinoma cell line (H1703, CRL5889) from the ATCC, and BLM cells [25]. Cell lines were cultured as indicated in Supplementary Table S1.

### Antibodies, reagents, cDNAs, western blotting, cell cycle and BrdU pulse/chase

Western blotting (WB) was performed as described [24] using the following antibodies: anti-p110α, -p110β, -phospho(p)-Akt S473, -p-Akt T308, -Akt total, -p-p70S6K T389, -PRAS40, -pERK T202/T204, and -ERK total (from Cell Signaling), anti-p85α (Millipore), anti-p70S6K (Santa Cruz), anti-p-PRAS40 T246 (R&D Systems), anti-α−tubulin (Calbiochem) and anti-β−actin (Sigma). We cloned human *PIK3R1* (p85α) or *PIK3R2* (p85β) shRNA into the Tet-pLKO-neo plasmid (a gift from Dimitri Wiederschain, Addgene 21916) [54]; shRNA expression from this vector is doxycycline-inducible (Sigma). 293T cells were transfected (JetPei DNA Transfection Reagent) with Tet-pLKO-neo-shp85α, Tet-pLKO-neo-shp85β or empty vector plus pMD2.G plasmid (Addgene 12259) and psPax2 plasmid (Addgene 12260) (48 h). Supernatants containing viral particles were filtered (0.45 μm), supplemented with polybrene (8 μg/ml, Sigma) and added to cells. After two infections, clones were selected in medium plus G418 (Calbiochem). Efficiency of shRNA induction was evaluated in WB; clones with >85% reduction of protein level were selected for *in vivo* and *in vitro* assays. PIK75 and TGX221 treatment, cell cycle analysis and BrdU pulse/chase were as described [39].

### Tumor xenografts

All procedures using mouse models were approved by the Ethics Committee of the Centro Nacional de Biotecnología (CNB-CSIC) and were carried in accordance with EU and Spanish legislation (RD 53/2013). Approximately 10^7^ cells (stably transfected with shRNA-p85α, -p85β or empty vector) were mixed (50%) with Matrigel (BD) in a total volume of 0.2 ml and were inoculated subcutaneously in the flank of 8-week-old female mice under isofluorane anesthesia. We used the *scid/scid* mouse strain (BALB/cJHanHsd-Prkdc^scid^), which develops neither T nor B cells, as well as (in the case of SK-MES and EPLC) the *scid/Beige* mouse strain (C.B-17/IcrHsd-Prkdc^scid^Lyst^bg-J^), which lacks T, B and NK cells (both strains from Harlan Laboratories).

Tumors developed for 3 to 7 days until the volume of most ranged from 75 to 100 mm^3^, after which we included doxycycline (2 mg/ml) in mouse drinking water containing 5% saccharose. We weighed mice and measured tumors with calipers twice weekly and calculated volume as V = (smaller side length^2^ x larger)/2. As endpoints, we established a) loss of 20% of initial weight, b) tumors greater than 1500 mm^3^ or c) complete loss of treated tumors for two weeks, indicating tumor regression.

### Histopathology, immunohistochemical analysis

Tissue samples for histopathology and immunohistochemistry were fixed in 10% buffered formalin, paraffin-embedded, sectioned (4 μm) and hematoxylin/eosin-stained. Indirect immunohistochemical staining was performed on formalin-fixed paraffin-embedded tissue sections, using streptavidin-biotin peroxidase complex [55]. Antibodies used for immunostaining were anti-Ki-67 (1:100; MIB-1, Dako) and -cleaved caspase 3 (1:100; 9661 rabbit IgG; Cell Signalling). Briefly, tissues were deparaffinized and sections hydrated in a graded ethanol series. Endogenous peroxidase activity was quenched with 3% hydrogen peroxide (10 min). In order to retrieve the Ki-67 and cleaved caspase 3 antigens, the tissues were placed in citrate buffer (10 mM, pH 6) or EDTA buffer (0.5 mM, pH=8) for 20 min, and sections were incubated with primary antibody (overnight, 4°C). After rinsing with Tris-buffered saline (TBS), sections were incubated with peroxidase-based EnVision complex (Dako). Peroxidase activity was demonstrated by diaminobenzidine staining. Finally, sections were washed in water, lightly hematoxylin-counterstained, dehydrated and mounted with DPX (distyrene, a plasticizer, and toluene-xylene). For controls, primary antibody was omitted or samples were incubated with an isotype control antibody. The percentage of positive cells were evaluated by scoring 10 randomly selected 40X fields a light microscopy (Nikon Y-THS).

### Statistical analyses and *in silico* studies

*PIK3R2* mRNA expression was analyzed in different human lung cancer types using Oncomine, as described (www.oncomine.org) and we represented data obtained form Bhattacharjee et al. 2001 [30]. Gel band intensity was quantitated with ImageJ software (NIH). Significance was calculated with ANOVA, Chi square and Student's *t* test using GraphPad Prism 5.0 (GraphPad Software).

## SUPPLEMENTARY MATERIALS FIGURES AND TABLES




