# Supplementary material for: Targeted depletion of PIK3R2 induces regression of lung squamous cell carcinoma
Source: Oncotarget. 2016 Nov 8;7(51):85063–78. doi: 10.18632/oncotarget.13195 (PMC5356720; doi:10.18632/oncotarget.13195)
Supplement: Supplementary file 1 [file oncotarget-07-85063-s001.pdf]

Targeted depletion of *PIK3R2* induces regression of lung squamous cell carcinoma

SUPPLEMENTARY FIGURES AND TABLES

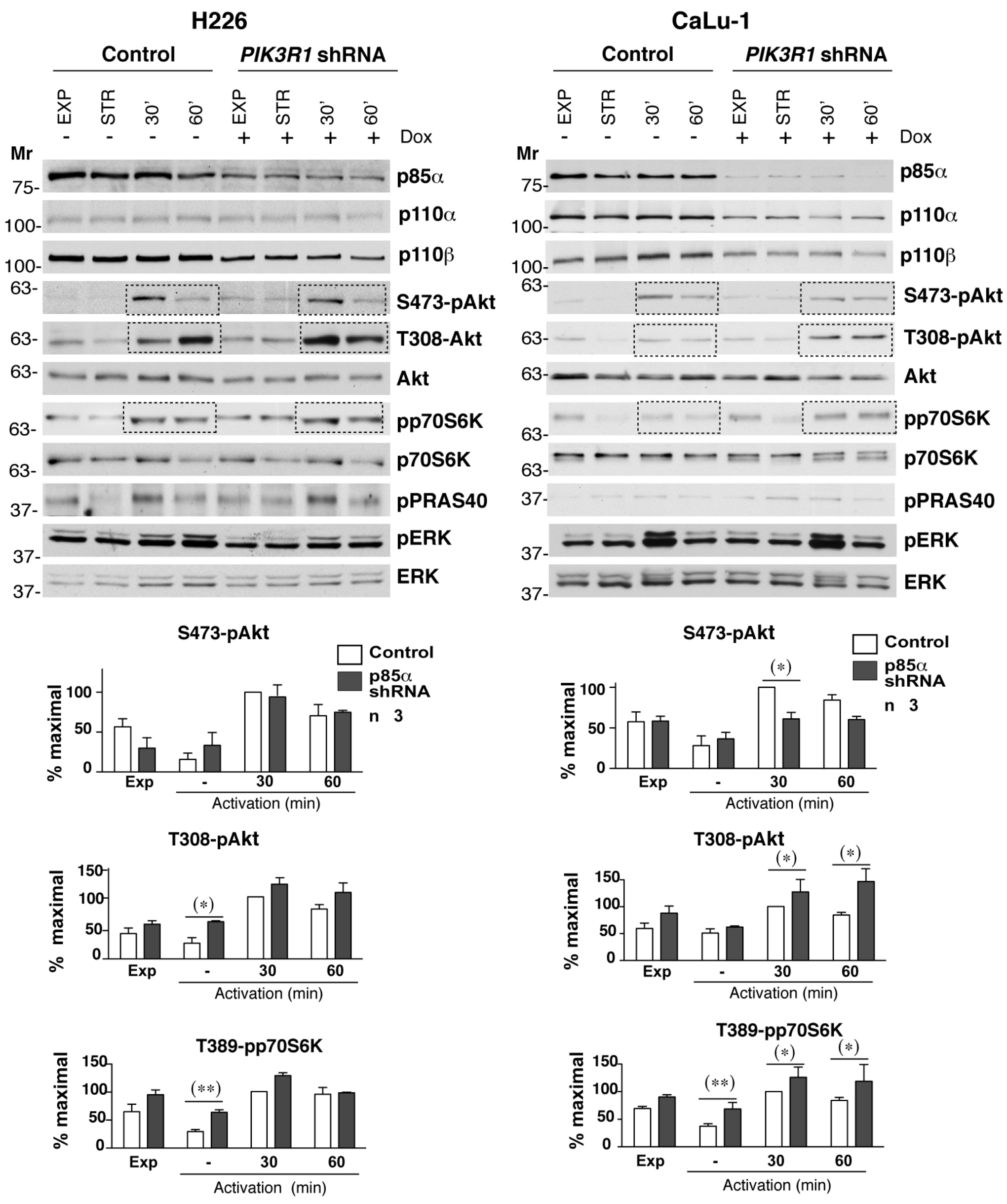

Supplementary Figure S1: p85α depletion enhances PI3K pathway activation in CaLu-1 and H226 cell lines. CaLu-1 or H226 cells expressing inducible PIK3R1 shRNA were cultured alone or with doxycycline (2 μg/ml, 72 h); extracts were analyzed in WB. Graphs show percentage of maximal S472-pAkt, T308-pAkt and T389-pp70S6K signal normalized to total Akt or p70S6K levels and compared to control cell signal at 30 min post-stimulation, considered 100%. * P <0.05, ** P <0.01; Student's t test.

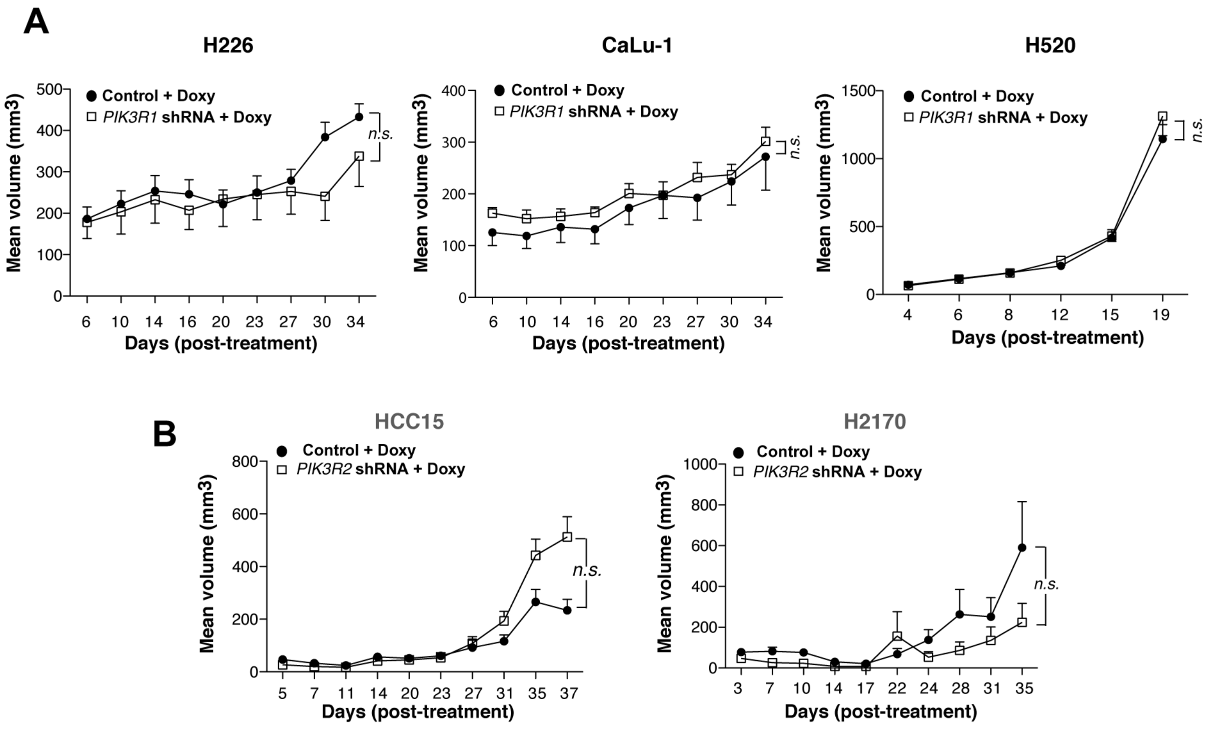

Supplementary Figure S2: PIK3R1 depletion does not trigger lung SQCC xenograft regression. A. The figure illustrates the response to PIK3R1 depletion of indicated tumor xenografts treated as in main Figure 3A and expressed as mean ± SEM size of the control or PIK3R1 shRNA-treated xenografts. **B.** Mean ± SEM size of control or PIK3R2 shRNA-treated xenografts as in main Figure 3B. A,B, Differences between control and treated mouse tumors was analyzed using a 2-way ANOVA test; n.s., not significant.

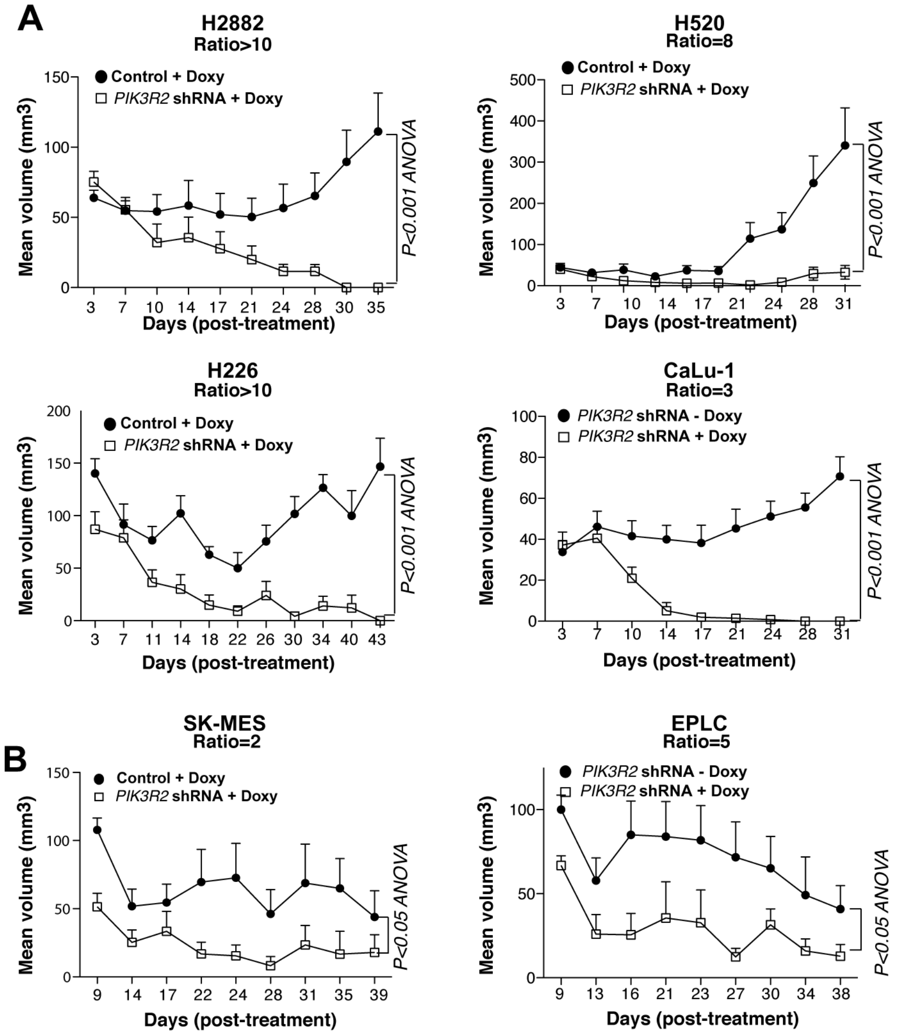

Supplementary Figure S3: PIK3R2 depletion induces SQCC xenograft regression. A. The figure illustrates the response to PIK3R2 depletion of indicated tumor xenografts treated as in main Figure 4A and expressed as mean ± SEM size of the control or PIK3R2 shRNA-treated xenografts. **B.** Mean ± SEM size of control or PIK3R2 shRNA-treated xenografts as in main Figure 4B. Statistical significance was analyzed using a 2-way ANOVA test.

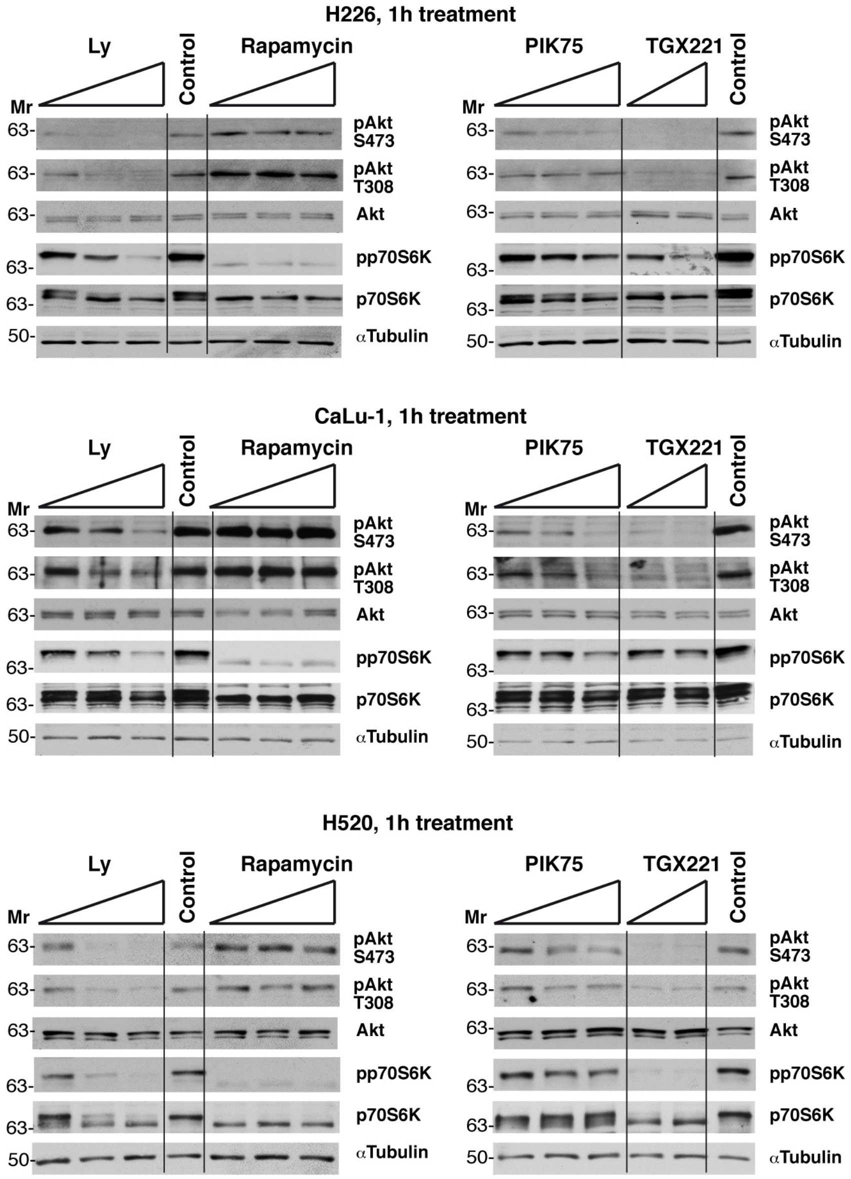

Supplementary Figure S4: Selective inhibitors for PI3Kα or PI3Kβ inhibited PI3K pathway at short times in SQCC cells. H226, CaLu-1 and H520 cells were cultured in exponential growth with vehicle (DMSO, 1:10^3 V:V) or Ly294002 (1, 5 or 10 µM), TGX221(15 or 30 µM), PIK75 (50, 100, or 200 nM), or rapamycin (25, 50, 100 nM) for the last 1 h of culture. Cell extracts were tested in WB with indicated antibodies. Mr indicates relative mobility.

Supplementary Table S1: Summary of tissue culture medium requirements of cell lines used

Cell line (ATTC/DSMZ reference)	Medium*	FBS	Other
CaLu-1 (ATCC HTB-54).	DMEM	10%	
SK-MES-1 (ATCC HTB-58).	EMEM	10%	
SW900 (ATCC HTB-59)	Leibovitz's L-15	10%	
NCI-H520 [H520] (ATCC HTB-182)	RPMI 1640	10%	
NCI-H226 [H226] (ATCC CRL-5826)	RPMI 1640	10%	
NCI-H1703 [H1703] (ATCC CRL-5889)	RPMI 1640	10%	
NCI-H1869 [H1869] (ATCC CRL-5900)	DMEM:F-12 (1:1)	10%	a) 0.02 mg/ml insulin<br>b) 0.01 mg/ml transferrin<br>c) 25 nM sodium selenite<br>d) 50 nM hydrocortisone<br>e) 1 ng/ml epidermal growth factor (EGF)<br>f) 0.01 mM ethanolamine<br>g) 0.01 mM phosphorylethanolamine<br>h) 100 pM triiodothyronine<br>i) 0.5% (w/v) bovine serum albumin<br>j) 10 mM HEPES<br>k) 0.5 mM sodium pyruvate
NCI-H2170 [H2170] (ATCC CRL-5928)	RPMI 1640	10%	
NCI-H2882 NCI-H2170 [H22882] (ref26)	RPMI 1640	10%	
EPLC-272H (ACC 383)	RPMI 1640	20%	
HCC-15 (ACC 496)	RPMI 1640	10%	
BLM (ref 25)	DMEM	10%	
Jurkat	RPMI 1640	10%	
MCF-10A	DMEM:F-12 (1:1)		a) 5% horse serum<br>b) 2 mM L-glycine<br>c) 10 µg/ml insulin<br>d) 0.5 µg/ml hydrocortisone<br>e) 20 ng/ml EGF

* All mediums were supplemented with 2 mM glutamine, 100 IU/mL penicillin and 100 µg/mL streptomycin
Abbreviations: DMEM: Dulbecco's modified Eagle's Medium; EMEM: Eagle's Minimum Essential Medium; FBS: fetal bovine serum (heat-inactivated); F-12: Ham's F-12 medium

Supplementary Table S2: Phenotype of SQCC cell lines studied. (Column 1) the SQCC lines used for the study, (column 2) ratio of p85β/p85α levels estimated as described (see text), (column 3) Positive (YES) or negative (NO) response of SQCC cell line-derived tumor xenografts to inducible *PIK3R2* shRNA treatment; N.D., not determined; (column 3) mutational status of indicated genes, (column 5) other potentially relevant mutations in these cells. (column 6) Rate of cell division in culture, which was unrelated to efficiency of xenograft establishment. +++, <1 week required for confluence recovery after 1:5 dilution; ++, 1 week to 10 days; +, 10 days to two weeks; ±, >2 weeks. (column 7) Relative *PIK3CA, CB, R1* and *R2* expression; larger font size indicates predominant isoforms

See Supplementary File 1
