# Supplementary material for: Targeted depletion of PIK3R2 induces regression of lung squamous cell carcinoma
Source: Oncotarget. 2016 Nov 8;7(51):85063–78. doi: 10.18632/oncotarget.13195 (PMC5356720; doi:10.18632/oncotarget.13195)
Supplement: Supplementary file 2 [file oncotarget-07-85063-s002.doc]

**Table S2. Phenotype of SQCC cell lines studied.**  (Column 1) the SQCC lines used for the study, (column 2) ratio of p85β/p85α levels estimated as described (see text), (column 3) Positive (YES) or negative (NO) response of SQCC cell line-derived tumor xenografts to inducible *PIK3R2* shRNA treatment; N.D., not determined; (column 3) mutational status of indicated genes, (column 5) other potentially relevant mutations in these cells. (column 6) Rate of cell division in culture, which was unrelated to efficiency of xenograft establishment. +++, <1 week required for confluence recovery after 1:5 dilution; ++, 1 week to 10 days; +, 10 days to two weeks; ±, >2 weeks. (column 7) Relative *PIK3CA, CB, R1* and *R2* expression; larger font size indicates predominant isoforms.

| **SQCC** | **p85α/p85β**  **RATIO** | **Tumor**  **Response** | **Mutations** | | | | **Other**  **mutations** | **Growth rate in vitro** | **PI3K subunits** |
| --- | --- | --- | --- | --- | --- | --- | --- | --- | --- |
|  |  |  | ***TP53*** | ***PIK3CA*** | ***KRAS*** | ***PTEN*** |  |  |  |
| **H2882** | **>10** | **YES** | WT | N.D. | N.D. | N.D. | RHOB**^1^** | **+** | **R2 CB** **CA** |
| **H226** | **>10** | **YES** | WT | WT | WT | WT | APC, MAP3K19, etc.**^2,3,5^** | **+++** | **R2 CB** |
| **H520** | **~8** | **YES** | Mut | WT | WT | WT | EGFR, ROCK2, etc. **^2,5^** | **+** | **R2 R1 CB** **CA** |
| **SW900** | **~6** | **N.D.^a^** | Mut | WT | Mut | WT | SMAD3, MAP3K4, etc. **^2^** | **+/-** | **R2 CB** **CA** |
| **EPLC272H** | **~6** | **YES** | Mut | WT | WT | WT | MAP4K4, RICTOR, NF1, etc. **^2^** | **+** | **R2 CB** **CA** |
| **CaLu-1** | **~3** | **YES** | WT | N.D. | Mut | WT promoter Me^4^ | EGFR, ROCK2, etc. **^2^**,**^5^** | **+++** | **R2 R1 CB** **CA** |
| **SK-MES** | **~2** | **YES** | Mut | WT | WT | WT promoter Me^4^ | ERBB4, LATS, NF1,TSC2,etc. **^2^** | **+++** | **R2 R1 CB** **CA** |
| **HCC15** | **~1** | **NO** | Mut | WT | WT,NRAS mut | WT | MET, EGFR, ABL, etc. **^2^** | **+++** | **R2 CB** **CA** |
| **H1869** | **~1** | **N.D.^b^** | Mut | WT | WT | WT | MET, FOXO1, RICTOR, etc. **^2^** | **+** | **R2 R1CB** **CA** |
| **H2170** | **~1** | **NO** | Mut | WT | WT | WT | PIK3CG, RHOA, etc. **^2,5^** | **+** | **CB** **CA** |

**^a^ Growth very slow ^1^ No COSMIC data; described in Cancer Res 2003, 63:6272; Int J of Cancer 2007,120:543.**

**http://igrcid.ibms.sinica.edu.tw/cgi-bin/cell_line_view.cgi?cl_name=H2882GEO.**

**^b^ Non-infectable ^2^ http://cansar.icr.ac.uk/xansar/cell-lines/**

**^3^ Cancer Res. 2011, 71: 7071.**

**^4^ Clin Cancer Res 2002, 8: 1178.**

**^5^ Hum Mutat. 2009, 30: 1199.**
